# Supplementary material for: Comparative characterization reveals conserved and divergent ecological traits of oral corynebacteria
Source: Microbiol Spectr. 2025 Dec 22;14(2):e02973-25. doi: 10.1128/spectrum.02973-25 (PMC12889116; doi:10.1128/spectrum.02973-25)
Supplement: Supplemental figures and table — Figures S1 and S2, and Table S1. [file spectrum.02973-25-s0001.pdf]

Supplemental Tables and Figures

Comparative Characterization Reveals Conserved and Divergent Ecological Traits of Oral Corynebacteria

Molly Burnside[1], Emily Helliwell[1], Puthayalai Treerat[1,2], Tanner Rozendal[3], Justin Merritt[1,4], Jonathon L. Baker[1], and Jens Kreth[1,4]

[1] Biomaterial and Biomedical Sciences, School of Dentistry, Oregon Health & Science University (OHSU), Portland, OR 97239, USA.

[2] Division of Infectious Diseases and International Health, Department of Medicine, University of Virginia, Charlottesville, Virginia, USA.

[3] Clark Honors College, University of Oregon, Eugene, Oregon, USA.

[4] Department of Molecular Microbiology and Immunology, School of Medicine, Oregon Health & Science University (OHSU), Portland, OR 97239, USA.

Strains	Characteristics	Reference
C. durum JJ1	Clinical isolate (GenBank accession number MN251472)	Treerat *et al*. 2020
C. matruchotii ATCC 14266	*C. matruchotii* reference strain	Barrett *et al*. 2001
C. matruchotii NCTC 10206	*C. matruchotii* reference strain #NC_003450	RefSeq: GCF_900638255.1
C. durum JJ2	Sequenced isolate (GenBank accession number CP198957)	this study
C. argentoratense MB1	Sequenced isolate (GenBank accession number)	this study
C. glutamicum ATCC 13032	*C. glutamicum* reference strain #NC_003450.3	RefSeq: GCF_000011325.1
S. sanguinis SK36	*S. sanguinis* wild type	Xu et al. 2007

Supplemental Table 1: Bacterial species used in this study.

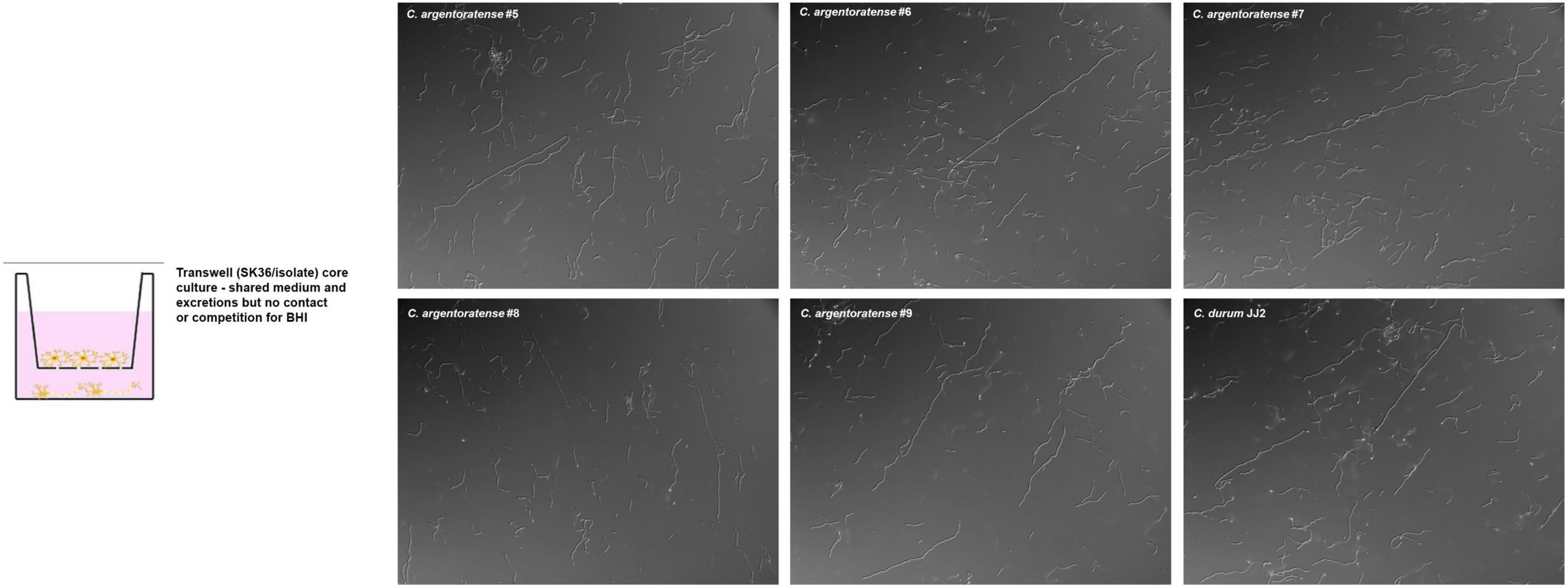

Supplemental Figure 1: Transwell assays for SK36 with *Corynebacterium argentoratense* #5, SK36 with *Corynebacterium argentoratense* MB1, *Corynebacterium argentoratense* #7, *Corynebacterium argentoratense* #8, *Corynebacterium argentoratense* #9, and *Corynebacterium durum* JJ2.

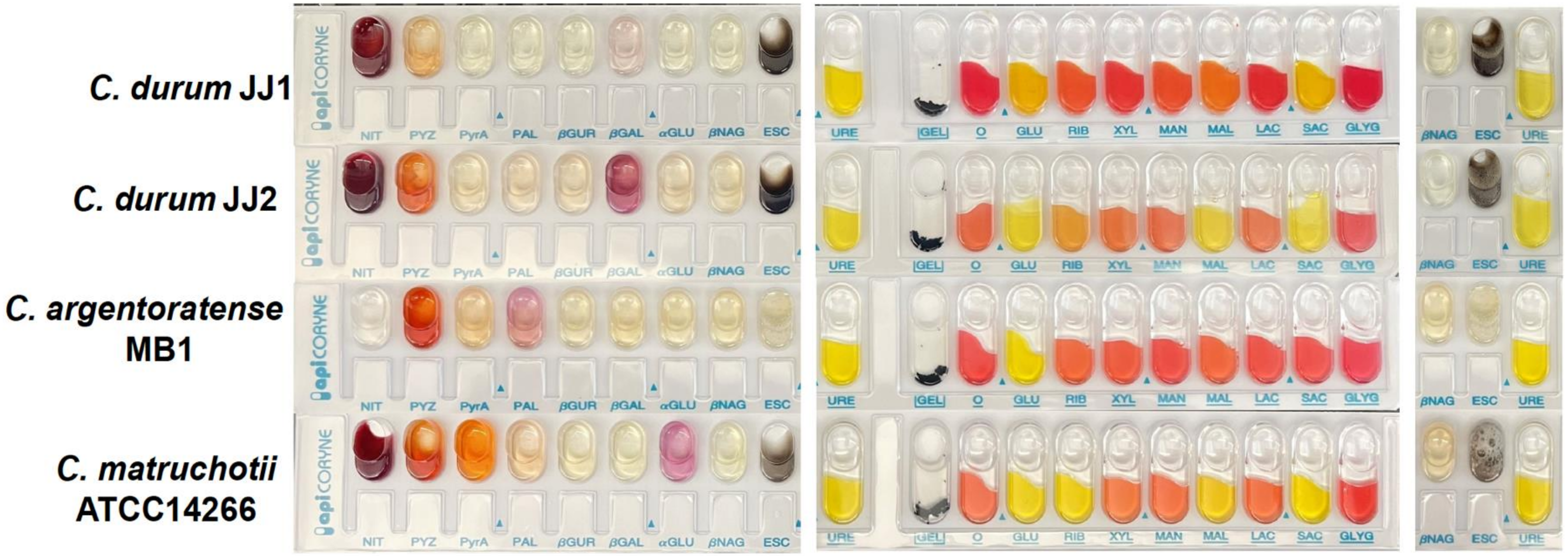

Strains	NIT	PYZ	PyrA	PAL	βGUR	βGAL	αGLU	βNAG	ESC	URE	GEL	O	GLU	RIB	XYL	MAN	MAL	LAC	SAC	GLYC	CAT
C. durum JJ1	4+	1+	–	–	–	–	–	–	3+	–	-	–	4+	1+	-	1+	2+	-	4+	-	+
C. durum JJ2	4+	3+	–	–	–	2+	–	–	4+	–	-	-	4+	2+	-	-	3+	-	4+	-	+
C. argentoratense MB1	–	4+	–	–	–	–	–	–	-	–	–	-	4+	–	–	–	–	-	–	–	+
C. matruchotii ATCC14266	4+	3+	4+	–	–	–	2+	–	2+	-	–	-	4+	4+	-	-	3+	-	4+	-	+

Supplemental Figure 2: Biochemical analysis of 4 *Corynebacterium* isolates using Biomeriux API® strips. A. Biochemical tests for the following enzymatic reactions: nitrate reduction (NIT), pyrazinamidase (PYZ), pyrrolidonyl arylamidase (PyrA), alkaline phosphatase (PAL), β-Glucuronidase (β-GUR), β-Galactosidase (β-GAL), α-Glucosidase (α-GLU), N-Acetyl-β-glucosaminidase (βNAG), β-Glucosidase (ESC). Color change indicates positive reaction. B. Biochemical tests for enzymatic reaction of urease (URE), hydrolysis of gelatin (GEL), and fermentation of sugars: glucose (GLU), ribose (RIB), xylose (XYL), mannitol (MAN), maltose (MAL), lactose (LAC), sacrose (SAC), and glycogen. Yellow/orange color indicates positive reaction, red color indicates negative reaction, O cupule used as negative control. C. Hydrogen peroxide test on β-Glucosidase (ESC). Formation of bubbles indicates positive reaction.
